# Histopathological evaluation of orbital and ocular lesions: A cross-sectional study

**DOI:** 10.6026/973206300221482

**Published:** 2026-03-31

**Authors:** Amit Prakash Jain, Manish Sangtani, Bhumika Sangtani, V.K Shrivastava

**Affiliations:** 1 Department of Pathology, District Hospital, Damoh, Madhya Pradesh, India; 2 Department of dentistry, District hospital, Damoh, Madhya Pradesh, India; 3 Department of Dentistry, Citysmile Dental Clinic, Damoh, Madhya Pradesh, India; 4 Department of Pathology, NSCB Medical College, Jabalpur, Madhya Pradesh, India

**Keywords:** Ocular lesions, orbital tumors, histopathology, eye pathology, cross-sectional study

## Abstract

Orbital and ocular lesions encompass a variety of inflammatory, congenital, benign and malignant conditions, often with overlapping clinical features that make diagnosis challenging. Therefore, it is of interest to investigate the histological spectrum of these lesions and correlate the findings with clinical diagnoses. It was conducted over 18 months in a tertiary care teaching hospital using histopathological examination of surgically removed specimens. Results showed that non-neoplastic lesions were most common, followed by benign and malignant tumors. This study advances knowledge by emphasizing the importance of histopathology as the gold standard for accurate diagnosis and treatment planning.

## Background:

Orbital and ocular lesions include a wide range of disease entities that affect the eyelids, conjunctiva, lacrimal apparatus, orbit and intraocular structures. These lesions may be congenital, inflammatory, degenerative, traumatic or neoplastic in nature and they can afflict people of any age. Because of the eye's functional and cosmetic importance, even minor abnormalities can cause severe morbidity if not detected and treated properly [1]. The initial stage in assessing ocular and orbital lesions is clinical evaluation, which involves a complete history, an ophthalmological examination and radiographic tests such as ultrasonography, computed tomography (CT) and magnetic resonance imaging (MRI). However, many lesions have overlapping clinical and radiological characteristics, making conclusive identification difficult on clinical grounds alone [2, 3]. As a result, histological analysis of removed tissue remains the gold standard for making an accurate diagnosis [4]. Ophthalmic pathology is critical in linking clinical symptoms with microscopic features, allowing for more precise classification of lesions and guiding suitable therapy options [5]. Histopathological examination is particularly crucial in discriminating between benign and malignant tumors, as clinically innocuous- appearing lesions can occasionally harbor malignancy [6]. Early detection of malignant ocular and orbital cancers considerably improves patient outcomes and lowers morbidity [7]. The orbit is a confined bony cavity containing vital neurovascular and muscular structures. Any pathological process within this space can rapidly lead to proptosis, visual impairment or optic nerve compression [8]. Similarly, lesions of the eyelid and conjunctiva are commonly encountered in routine clinical practice due to their exposed location, but their histopathological spectrum varies widely across different populations and geographical regions [9]. Several studies have found global variations in the occurrence and pattern of ocular and orbital lesions, which are influenced by genetic, environmental and socioeconomic variables [10]. Therefore, it is of interest to examine the histological spectrum of orbital and ocular lesions in a tertiary care hospital and connect histopathological findings with clinical diagnosis.

## Methodology:

A cross-sectional observational study was conducted over a period of 18 months (from 1st March 2016 to 31st August 2017) in the Department of Pathology, Netaji Subhash Chandra Bose Medical College, Jabalpur, a tertiary care teaching hospital. The inclusion criteria were surgically excised ocular and orbital lesions received during the study period from patients of all age groups and both sexes, provided adequate tissue was available for histopathological examination. Exclusion criteria included poorly preserved or autolyzed specimens, inadequate tissue samples and cases with incomplete clinical details. Specimens were fixed in 10% formalin and after thorough gross examination, representative tissue sections were processed routinely, embedded in paraffin and sectioned at 4-7 µm thickness. Hematoxylin and eosin staining was performed on all cases. Data were entered and analyzed using descriptive statistical methods, with results expressed as frequencies and percentages. Categorical variables such as age group, sex, anatomical site, nature of lesion and clinico-pathological correlation were analyzed using simple proportion analysis and presented in tables and graphs, with no inferential statistical tests applied due to the descriptive nature of the study.

## Results:

In this study, fifty cases of ocular and orbital lesions were evaluated for various clinico-pathological findings. The distribution of the cases across different genders and age groups is shown in Table 1 (see PDF). The most commonly involved location for the lesions was the eyelid, followed by intraocular lesions, as detailed in Table 2 (see PDF). Further breakdown of the eyelid lesions is provided in Table 3 (see PDF), which shows the specific locations within the eyelid, with the right upper eyelid being the most commonly affected. The correlation between clinical and histopathological diagnoses, as shown in Table 4 (see PDF), indicates that malignant lesions had a higher clinico-pathological correlation compared to benign lesions. The prevalence of various benign lesions is illustrated in Table 5 (see PDF), where cystic lesions were found to be the most common. Figure 1 (see PDF) illustrates the different types of eyelid lesions diagnosed in the study subjects, while Figure 2 (see PDF)displays the distribution of malignant lesions in the study cases.Among the benign lesions highest prevalence was of cystic lesions (38.46%) which included epidermal cyst, dermoid cyst, sebaceous cyst, sudoriferous cyst and mucocoele. Epidermal cyst was the predominant lesion amongst them. Cystic lesions were followed by infective lesions, non-specific inflammatory lesions and benign tumors in prevalence.

## Discussion:

The current investigation examined 50 cases of ocular and orbital lesions, revealing a broad histological spectrum including several anatomical regions of the eye and its adnexa. Previous studies have consistently reported such heterogeneity, reflecting the ocular and orbital regions' complex architecture and variable tissue composition [11]. In the current study, the maximum number of instances was found in the 1-10 age groups, followed by the 61-70 age groups. Previous researches have also shown a bimodal age distribution, with pediatric case predominately malignant and benign lesions more common in older age groups. The majority of pediatric instances are due to intraocular malignancies such as retinoblastoma, which usually appears in early childhood [12]. The male predominance (58%) seen in this study is consistent with findings reported by previous studies which also found a higher prevalence of ocular and adnexal lesions in men. However, the gender distribution varied throughout age groups, implying that gender inclination is driven by lesion type and age rather than representing a consistent trend. In terms of anatomical distribution, eyelid lesions were the most common, followed by intraocular abnormalities. Similar findings have been observed in several investigations, which ascribe the higher prevalence of eyelid lesions to their visible location and ease of clinical diagnosis [13]. Upper eyelid involvement was more common than lower eyelid involvement, which could be attributed to the larger surface area and density of adnexal structures in the upper eyelid. Benign lesions predominated in the current study, accounting for 78% of cases. Cystic lesions were the most prevalent among benign lesions, with epidermal cysts being the most common subtype. This pattern has been described repeatedly in previous investigations, underlining the prevalence of cystic and inflammatory lesions in ordinary ophthalmology practice [14]. Infective and non-specific inflammatory lesions accounted for a large proportion, emphasizing the importance of chronic inflammation and local infections in ocular pathology. Malignant lesions accounted for 22% of patients in the current investigation. Retinoblastoma was the most prevalent malignant tumor, particularly in the pediatric population. This finding is consistent with worldwide and Indian researches, which have identified retinoblastoma as the most frequent intraocular cancer in children [15]. The early age of presentation reported in this study adds to the congenital and hereditary foundation of this disease. Other malignant lesions found included squamous cell carcinoma and basal cell carcinoma, which primarily affected the eyelid. Similar patterns have been observed in research concentrating on ocular adnexal malignancies, with UV exposure, persistent irritation and delayed presentation identified as important factors [16]. Clinicopathological association was detected in 54% of cases, with malignant lesions having a stronger correlation than benign lesions. This higher correlation in malignant lesions could be attributed to their more unique clinical and radiological characteristics, which aid in clinical suspicion and diagnosis. In contrast, benign lesions frequently have overlapping clinical presentations, creating diagnostic confusion without histological confirmation. Overall, the findings of this study emphasize the importance of histological evaluation in the identification of ocular and orbital lesions. All removed specimens must be routinely submitted for histological investigation to ensure accurate diagnosis, discover clinically unexpected cancers and advise proper patient management [17].

## Conclusion:

We show the diverse histological spectrum of ocular and orbital diseases, with benign lesions, particularly cystic ones, being more common than malignant tumors. The eyelid was the most frequently affected site and retinoblastoma was the most common malignant tumor, especially in children. Routine histological examination is crucial for accurate diagnosis, early cancer detection and optimal patient care.

## References

[1] Mafee MF (1996). Neuroimaging Clin N Am..

[2] Rootman J (2014). Ophthalmic Plast Reconstr Surg..

[3] Lanni V (2021). J Ultrasound..

[4] Ho M (2013). Hong Kong Med J..

[5] Nuessle S (2026). BMC Ophthalmol..

[6] Ohshika S (2021). Oncol Lett..

[7] Ma J (2024). Can J Ophthalmol..

[8] Kalentakis Z (2025). Sinusitis,.

[9] Banerjee et (2022). Indian J Ophthalmol..

[10] Goyal S (2025). World J Clin Pediatr..

[11] Adamski WZ (2021). Postepy Dermatol Alergol..

[12] https://www.ncbi.nlm.nih.gov/books/NBK582155/.

[13] Nag A, Khetan V (2024). Indian J Ophthalmol..

[14] Al Wohaib M (2018). Middle East Afr J Ophthalmol..

[15] Kwee RM, Kwee TC (2019). Skeletal Radiol..

[16] Adile V (2025). MGM Journal of Medical Sciences.

[17] Rajbhar R (2020). Int J Health Sci (Qassim)..

